# A highly miniaturized circularly polarized self-duplexing implantable antenna with enhanced performance for wireless capsule endoscopy applications

**DOI:** 10.1038/s41598-025-19415-7

**Published:** 2025-10-10

**Authors:** Syed Misbah Un Noor, Syed Ahson Ali Shah, Izaz Ali Shah, Shahid Khan, Jamal Nasir, Slawomir Koziel, Qammer H. Abbasi

**Affiliations:** 1 Department of Electrical and Computer Engineering, COMSATS University Islamabad, Abbottabad Campus, Pakistan; 2 Department of Electrical Engineering and Computer Science, Gwangju Institute of Science and Technology, Gwangju, 61005 South Korea; 3 Department of Electronic Engineering, Hanyang University, Seoul, 04763 South Korea; 4 Faculty of Electronics, Telecommunication and Informatics, Gdansk University of Technology, Gdansk, 80-233 Poland; 5 Department of Engineering, Reykjavik University, Reykjavik, 102 Iceland; 6 James Watt School of Engineering, University of Glasgow, Glasgow, G12 8QQ UK; 7 Artificial Intelligence Research Centre, Ajman University, Ajman, United Arab Emirates

**Keywords:** Engineering, Gastroenterology

## Abstract

Multiband implantable antennas are crucial components of biomedical implantable devices (BIDs), enabling the establishment of wireless communication links with external base stations. These types of antennas perform various functions such as data transmission, wireless power transfer, and control signaling. However, this scenario requires an external multiplexer in the BIDs to separate various frequency bands, imposing size constraints on the BIDs. This work proposes a self-duplexing circularly polarized implantable (SDCPI) antenna for wireless capsule endoscopy (WCE) application having two separate ports, with port 1 providing a wideband response covering MICS (402 MHz), ISM (433 MHz) band, and port 2 covering ISM (915 MHz) band. The proposed SDCPI antenna achieved a very compact volume of π × (5.1)^2^ × 0.127 = 10.3 mm^3^ by semi-circular slots on the radiating patch and shorting pins. The proposed capsule integrated implantable antenna was thoroughly analyzed through simulations in various parts of the digestive tract (stomach, small intestine, and colon) and was later fabricated and tested. The measurements were carried out in minced pork, and the results obtained showed close resemblance to the simulated results. The proposed SDCPI antenna offers a -10 dB impedance bandwidth and CP bandwidth of 151 and 273 MHz, and 10% and 15% at 402 and 915 MHz, respectively. Furthermore, it exhibits measured gain of -36 and − 25 dBi at 402 and 915 MHz, respectively. To evaluate the human safety of the proposed SDCPI antenna, specific absorption rate (SAR) at 402 and 915 MHz was estimated in the stomach, small intestine, and colon, and was found to be within the limits allowed by the IEEE standards. Additionally, the wireless communication link establishment capabilities of the proposed SDCPI antenna were gauged through link margin analysis. This analysis confirmed that at 402 MHz, the presented SDCPI antenna can establish reliable communication up to 25, 9.7, and 4.5 m when placed in the small intestine for bit rates of 1, 12, and 78 Mbps, respectively. Likewise, at 915 MHz, the suggested SDCPI antenna offers seamless communication up to 28, 14.7, and 5.3 m when placed in the small intestine for bit rates of 1, 12, and 78 Mbps, respectively. These results verify that, to the best of the authors’ knowledge, the proposed highly miniaturized SDCPI antenna is the first self-duplexing CP implantable antenna for WCE applications offering simultaneous transmission and reception without requiring an external multiplexer.

## Introduction

Advancements in biomedical implantable devices (BID) have played a vital role in the improvement of healthcare facilities worldwide^1^. This leads to the early detection of medical conditions, helping providers to increase the effectiveness of the healthcare system. These BIDs are crucial for various applications such as leadless cardiac pacemakers^2^, intraoral drive systems^3^, blood pressure monitoring^4,5^, glucose monitoring^6^, and wireless capsule endoscopy^7^. BIDs collect data from inside the body and transmit it to an off-body or on-body receiver. To establish the wireless communication link, an implantable antenna is an important component of a BID^8^. Implantable antennas, when compared to free space antennas, have entirely different characteristics. The size of an implantable antenna decides the size of the BID. Consequently, miniaturized implantable antennas are highly desired for BIDs^8^. In addition to antenna size, the operating bandwidth, patient safety quantified through specific absorption rate (SAR), body tissue coupling, and biocompatibility must be considered while designing an implantable antenna for specific applications^9^.

Recently, a variety of miniaturized implantable antennas featuring a single band^8,10,11^, dual-band^12–14^ and multiband operation^9,15,16^ have been suggested for various biomedical applications. In^8^, the authors presented a meandered line-based miniaturized implantable antenna for GHz ISM band operation with a compact volume of 4.42 mm^3^. Another single band implantable antenna based on meandered line geometry for operation in 401 − 406 MHz, Medical Implant Communication Service band (MICS) featuring a volume of 92.16 mm^3^, was studied in^10^. Furthermore, a dual-band implantable antenna operating in the 1.4 GHz Wireless Medical Telemetry Service (WMTS) and 2.45 GHz ISM band with a volume of 15.87 mm^3^ was proposed in^12^. The miniaturized size is achieved by using a high permittivity substrate and an open-end slot in the ground plane. Another dual-band implantable antenna operating in the ISM 0.9 and 2.45 GHz bands is proposed in^14^. The antenna has a small footprint of 92.16 mm^3^, achieved by incorporating shorting pins and the slotted ground plane in the design. Furthermore, a quad-band implantable antenna was suggested in^9^ operating in the 0.9, 2.45 GHz ISM band and 1.4, 1.9 GHz midfield band for wireless power transfer, control signaling, and data transfer applications. The design has a size of 4 mm × 3.5 mm × 0.05 mm, achieved using a shorting pin and the slotted ground plane.

All the implantable antennas discussed above exhibit a compact size and are proposed for a variety of biomedical applications with single-, dual-, and multiband functionalities. However, their single-port configuration restricts their simultaneous transmission and reception capabilities. To support the simultaneous transmission and reception operation, a multiplexer must therefore be incorporated into the BID, which poses a great challenge to be accommodated in the limited size of the BID. Besides, an increase in size results in an increase in the complexity and power consumption of the BID, greatly reducing the battery backup time^17^. To tackle this issue, the authors in^17–20^ proposed self-duplexing implantable antennas, which are capable of dual-mode operation (transmission and reception) without requiring a multiplexing circuit. In^18^, a self-duplexing implantable antenna with dual-band characteristics (915 and 1300 MHz) was proposed for wireless power and data telemetry applications in ingestible capsules with inter-port isolation of about 30 dB. The design has a compact volume of 8 × 8 × 0.13 mm^3^. Similarly, the authors in^19^ proposed a multipurpose biomedical implantable self-duplexing antenna for 915 and 1470 MHz bands occupying a volume of (4.8)^2^ × 3.14 × 0.13 mm^3^ and inter-port isolation of 21 dB. Another self-duplexing implantable antenna for dual-band operation (915 and 1420 MHz) was proposed in^17^ for head implant applications. The design occupies a volume of 7.13 × 8.9 × 0.13 mm^3^ and offers a port-to-port isolation of 31.4 dB. Furthermore, in^20^, a dual-band (915 and 1300 MHz) self-duplexing implantable antenna was proposed for simultaneous information delivery and wireless power transfer (WPT) applications. The antenna was proposed for pacemaker application and occupied a volume of π × 4.48^2^ × 0.13 mm^3^ with port-to-port isolation of 34.6 dB.

All the self-duplexing implantable antenna designs discussed above targeted the lower ISM band of 915 MHz and the mid-field bands of 1300 and 1400 MHz. The use of high-frequency bands results in a compact antenna design at the cost of lower EM tissue penetration depth due to the shorter wavelength. Additionally, the self-duplexing antennas discussed above are linearly polarized (LP) at both frequency bands, which is not ideal for implantable applications. Circularly polarized (CP) antennas, on the other hand, are highly preferred for reliable implantable communication with external receivers, as they effectively counteract postural changes and human movements^21^ and effectively mitigate multipath losses as compared to LP implantable antennas^22^. Considering the mentioned prerequisites, this work suggests an innovative design of a self-duplexing circularly polarized implantable (SDCPI) antenna for ISM bands (433.05-434.79 MHz, 902–928 MHz), and MICS band (402–405 MHz) for wireless capsule endoscopy (WCE) applications. The main contributions of this work are as follows:

- The development of a novel CP self-duplexing implantable antenna with one port resonating at the MICS band (402 MHz) and the lower ISM band (433 MHz) while the other port resonating at the upper ISM band (915 MHz).
- Achieving compact design by using a slotted circular patch and slots in the ground plane.
- Ensuring low SAR and acceptable link budget performance.

The rest of the article is organized as follows: Section II presents the proposed methodology for designing of the SDCPI antenna, along with simulations and the measurement setup. Section III includes parametric analysis. Section IV examines the antenna’s performance metrics, including the reflection coefficient, radiation patterns, SAR, and link budget analysis. Section V concludes the work.

## Design methodology

### Antenna and IMD geometry

**Fig. 1:**
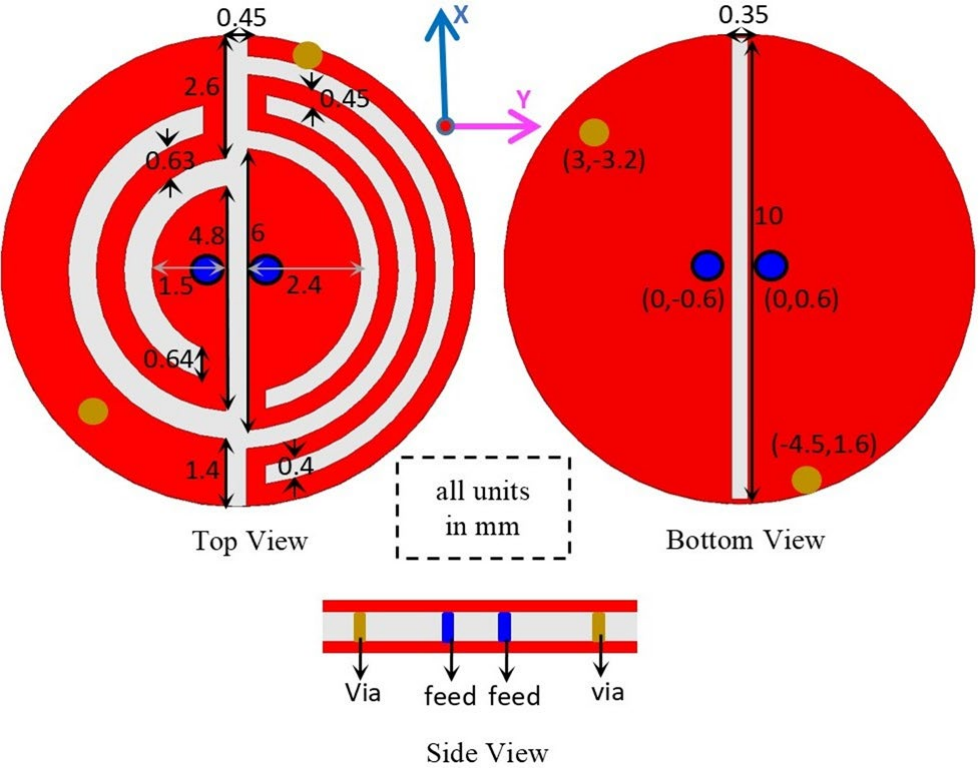
Geometry of the proposed SDCPI antenna.

The top, bottom, and side views of the presented SDCPI antenna are illustrated in Fig. 1. It consists of two radiating semi-circular patches etched on the top side of a 0.127-mm-thick Rogers RO3010 substrate (*ε*_*r*_ = 10.2 and tanδ = 0.002) backed by a ground plane. The radiating patches are loaded with open-ended arc-shaped slots and shorting pins (vias) to extend the current path and achieve size miniaturization, resulting in a compact volume of π × (5.1)^2^ × 0.127 = 10.3 mm^3^. The radiating patch on the right-hand side is loaded with more slots (to resonate at 403 MHz) as compared to the patch on the left-hand side (to resonate at 915 MHz). The ground plane has a slot (10 mm × 0.35 mm) to adjust the axial ratio (AR) bandwidth and reduce mutual coupling between the ports. Both the radiating patches are excited by 50 Ω probes as shown in Fig. 1. The positions of the feeding probes and shorting pins are optimized to obtain the best possible results.

### Simulation and measurement environments

**Fig. 2:**
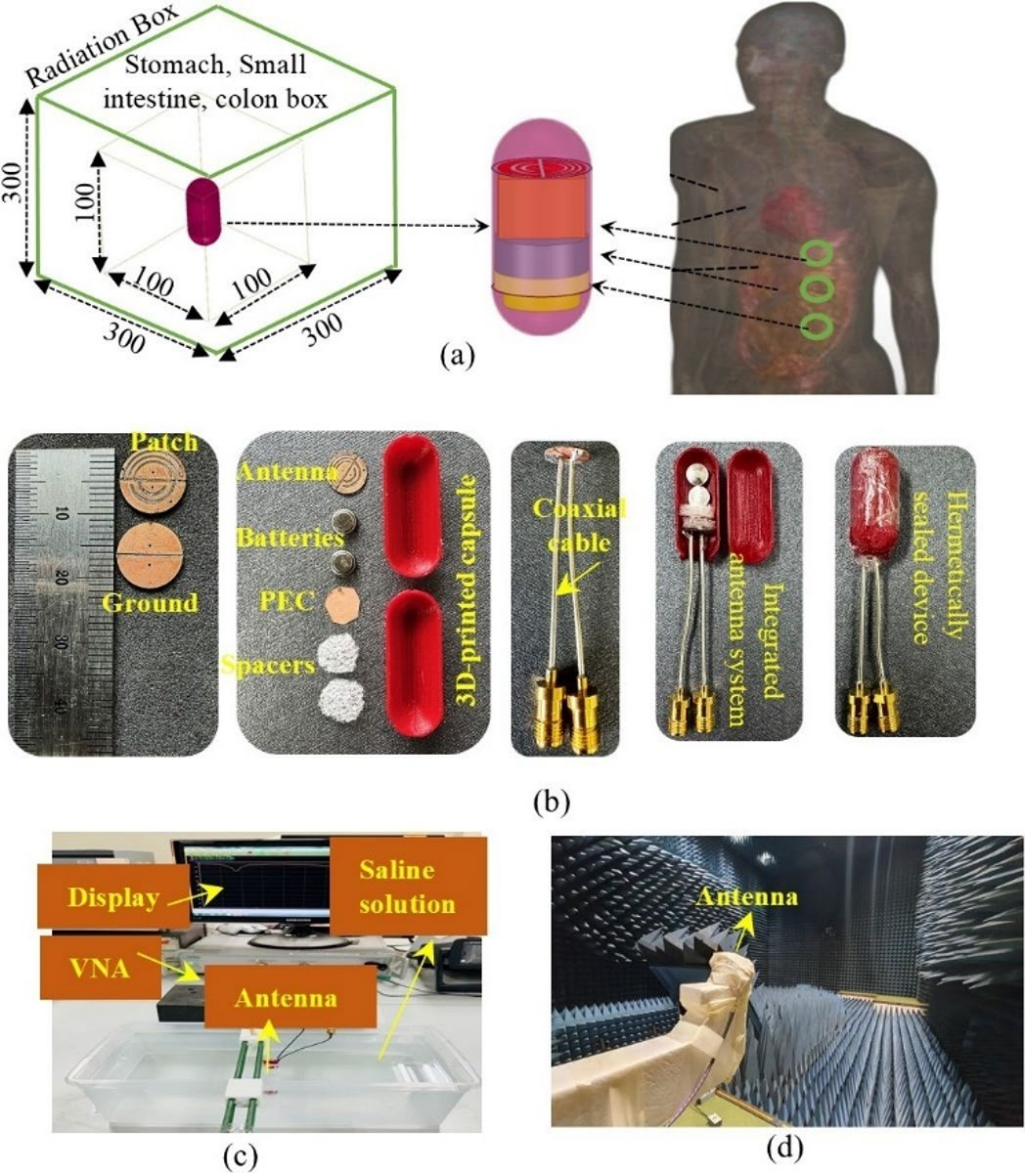
Proposed SDCPI antenna (**a**) Simulation setup, (**b**) Fabricated prototypes, and (**c**) S-parameters and radiation pattern setups.

Implantable antennas are placed inside the human body to transmit various information to an outside base station. Therefore, the setting up of an appropriate simulation environment is very important. As the proposed SDCPI antenna is intended for WCE application, an appropriate simulation environment has been considered. The antenna was initially simulated in homogeneous stomach, small intestine and colon phantoms having volumes of 100 mm × 100 mm × 100 mm inside FEM based HFSS simulation tool. To mimic the real-life scenario, the proposed SDCPI antenna was integrated inside a capsule-type BID and placed at a depth of 20 mm inside the homogeneous phantoms. The frequency-dependent properties of the stomach, small intestine, and colon are presented in Table 1^23–25^. The homogeneous phantoms were surrounded by the 300 mm × 300 mm × 300 mm radiation boundary as shown in Fig. 2a. Subsequently, the proposed SDCPI antenna was simulated using the Sim4Life EM tool to evaluate the impact of a heterogeneous environment and calculate the specific absorption rate (SAR), as illustrated in Fig. 2a.

The prototype of the proposed SDCPI antenna was fabricated was fabricated using Rogers RO3010 (*ε*_*r*_ = 10.2, tanδ = 0.0035, and height *h* = 0.5 mm) and enclosed inside a 3-D printed capsule containing batteries and PCB to validate the simulation results, as shown in Fig. 2b. The capsule shell was made up of biocompatible polylactic acid (PLA). The capsule was sealed with epoxy to make it leak-proof and durable. The measurement setup for *S*-parameters and radiation patterns is depicted in Fig. 2c, d. For the *S*-parameters measurement, the proposed SDCPI antenna was placed inside saline based solution, and both of its ports were connected to a fully calibrated vector network analyzer (VNA). Similarly, to measure the radiation patterns, the proposed SDCPI antenna was placed inside a container filled with minced pork and placed inside an anechoic chamber. The variations in pork tissue composition, such as fat content, moisture, and heterogeneity, may introduce minor differences in measured parameters compared to standardized human tissue phantoms. To mitigate these effects, a low-fat, homogenous batch of minced pork was used consistently across all measurements. Furthermore, the experimental results were cross-validated with full-wave simulations using heterogeneous realistic and frequency-dependent human tissue models (stomach, colon, small intestine, and heart) implemented in HFSS. One port of the presented SDCPI antenna (acting as a receiving antenna) was connected to the VNA, whereas the other port was terminated using a matched load. The second port of the VNA was connected to a standard horn antenna (acting as a transmitter). The same procedure was adopted for the other port.

### Design evolution

**Fig. 3:**
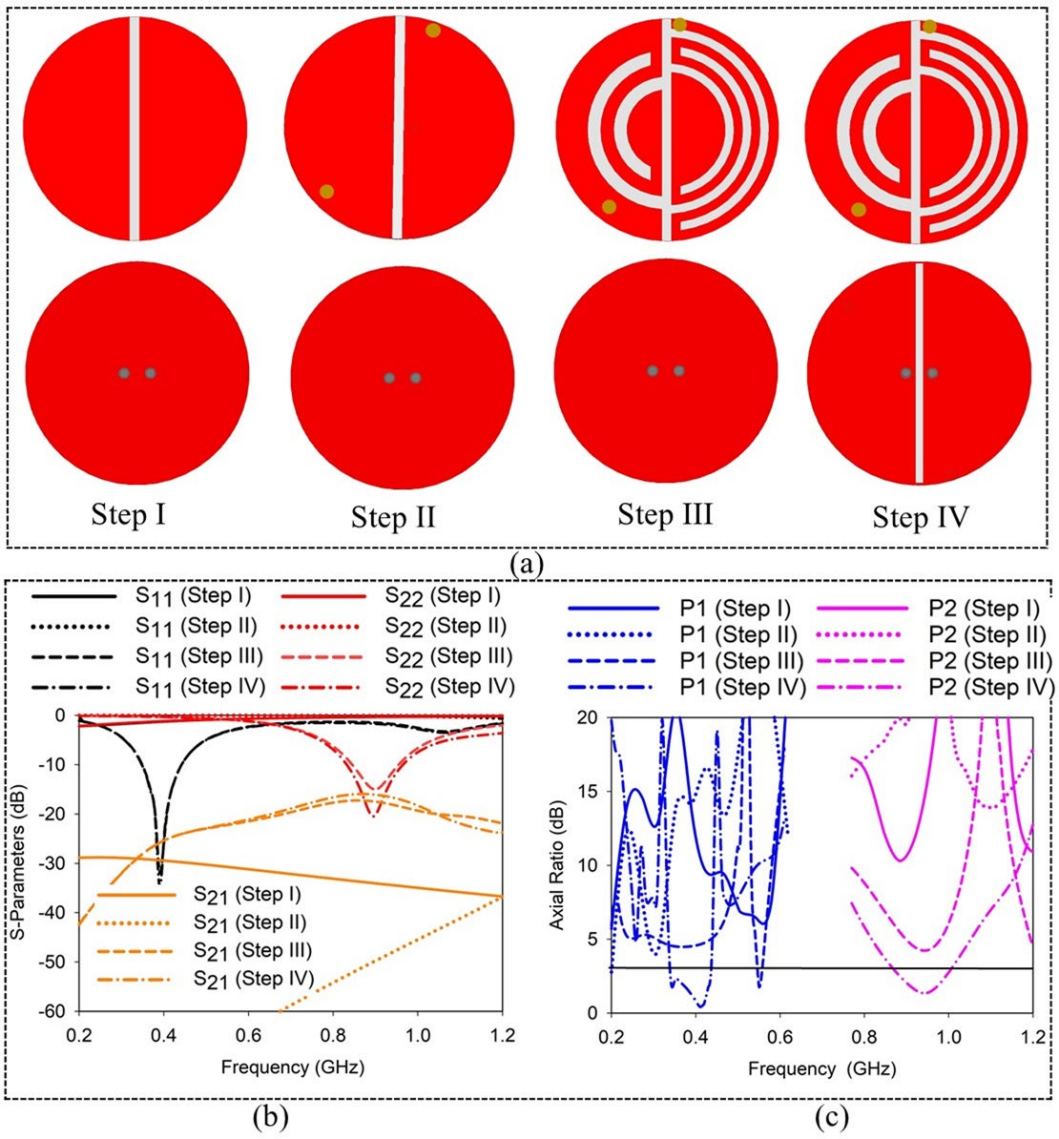
Proposed SDCPI antenna (**a**) Design steps, (**b**) S-parameters, and (**c**) AR of the proposed SDCPI antenna.

The design evolution process of proposed SDCPI antenna is presented in Fig. 3a. The corresponding *S*-parameters (S_11_, S_21_, S_22_) and AR (for port 1 and port 2) are presented in Fig. 3b, c, respectively. The initial radius of the circular radiating patch is obtained using the following formula^19^, and further optimized to obtain the desired results

1 $$\:{f}_{0}=\frac{1.841\times\:c}{4\pi\:R\sqrt{{\epsilon\:}_{r}}}$$

In (1), *f*_0_ is the resonance frequency, c = 3.8 × 10^9^ m/s is the speed of light, *R* is radius of the radiating patch, whereas *ε*_*r*_ is the substrate permittivity. The proposed SDCPI antenna evolved in four steps, and the position of the feeding probes in each step remains fixed. The design process is discussed below.

Step I: Using (1), the initial radius of the circular patch was found to be 5.1 mm to realize a resonance at the desired frequency. A circular radiating patch of *R* = 5.1 mm was etched on the top side of the substrate with a ground plane on the bottom side. Further, the radiating patch was split into two halves by a rectangular slot to accomplish a self-duplexing antenna as shown in Fig. 3a. Two feeding probes were used to excite each radiating part. The corresponding *S*-parameters plot in Fig. 3b shows no resonance in the band from 0 GHz to 1.2 GHz. The AR (Fig. 3c) in the targeted band of 402 and 915 MHz remains well above 3 dB.

Step II: To bring the resonance frequencies of the radiating patches towards the desired bands, shorting pins were introduced as shown in Fig. 3a. The shorting pins introduce more inductive effects, resulting in lowering the resonance frequencies, altering the current distribution and achieving size miniaturization. This arrangement results in the creation of two weak resonances (one for each radiating patch) around 1.2 GHz, as indicated in Fig. 3b. The mutual coupling between the patches remains low. The AR plot in Fig. 3c confirms that the AR still remains well above 3 dB.

**Table 1:** Electrical properties of various GI tract tissues at 2.45 GHz.

Parameters	Stomach	Small intestine	Colon
Permittivity (εr)	62.2	54.4	53.9
Conductivity (σ, S/m)	2.21	3.17	2.04

Step III: To achieve impedance matching in the bands of interest (402 and 915 MHz), arc-shaped slots of various dimensions were added to both radiating patches as depicted in Fig. 3a. To implement a resonance at 402 MHz, the slots of longer lengths were introduced to the patch on the right side as compared to the patch on the left side, which resonates at 915 MHz. The incorporation of the slots results in increasing the capacitive effect, which, in turn, results in lowering the resonances and achieving miniaturization according to^20^

2 $$\:{v}_{pr}=\frac{1}{\sqrt{LC}}={f}_{0}\lambda\:$$

where *v*_*pr*_ is the propagation velocity, *L* is the equivalent inductance, *C* is the equivalent capacitance, *f*_0_ is the resonance frequency, and *λ* is the wavelength. Equation (2) indicates that the increased inductive or capacitive effect results in lowering the resonance frequency. Figure 3b shows that the introduction of the slots to the patches results in lowering the resonance frequencies of Step II to the desired 402 and 915 MHz bands. It also results in the creation of near-orthogonal field components. The isolation between the ports remains above 18 dB in the bands of interest. However, the AR plot in Fig. 3c reveals that the AR in both bands is still above 3 dB as the E-field components are not in complete quadrature arrangement. Thus, further improvements are required.

Step IV: To reduce the AR in the desired bands below 3 dB, a rectangular slot was etched on the ground plane. This slot alters the current distribution beneath the radiators and enhances the orthogonality of the electric fields, introducing the required 90° phase difference between them. As a result, the antenna achieves circular polarization with AR < 3 dB at both operating bands. Additionally, the matching of the first resonance remains unaffected while that of the second resonance improves as indicated in Fig. 3b. However, the isolation in the upper band decreases to about 15 dB. Furthermore, AR at both bands improves significantly and remains below 3 dB (337 ‒ 434 MHz) in the lower band and (864 ‒1000 MHz) in the upper band as clear from Fig. 3c. The proposed SDCPI antenna design operates at MICS (402 MHz) band, the lower (433 MHz) ISM band with a bandwidth of 92 MHz (352 ‒ 444 MHz), and the upper ISM (915 MHz) band with a bandwidth of 147 MHz (825 ‒ 972 MHz). The 3-dB AR bandwidth at the MICS, lower ISM band is 97 MHz (337 ‒ 434 MHz), whereas at the upper ISM band it is 136 MHz (864 ‒1000 MHz).

### Parametric analysis

It is critical to analyze the effects of various design parameters on the performance of an implantable antenna to achieve an optimum design. In this section, the effects of the feeding probe position, radius of the shorting pins, and the location of the SDCPI antenna inside the capsule are carefully analyzed in terms of *S*-parameters and AR.

#### Effect of feeding probe positions

**Fig. 4:**
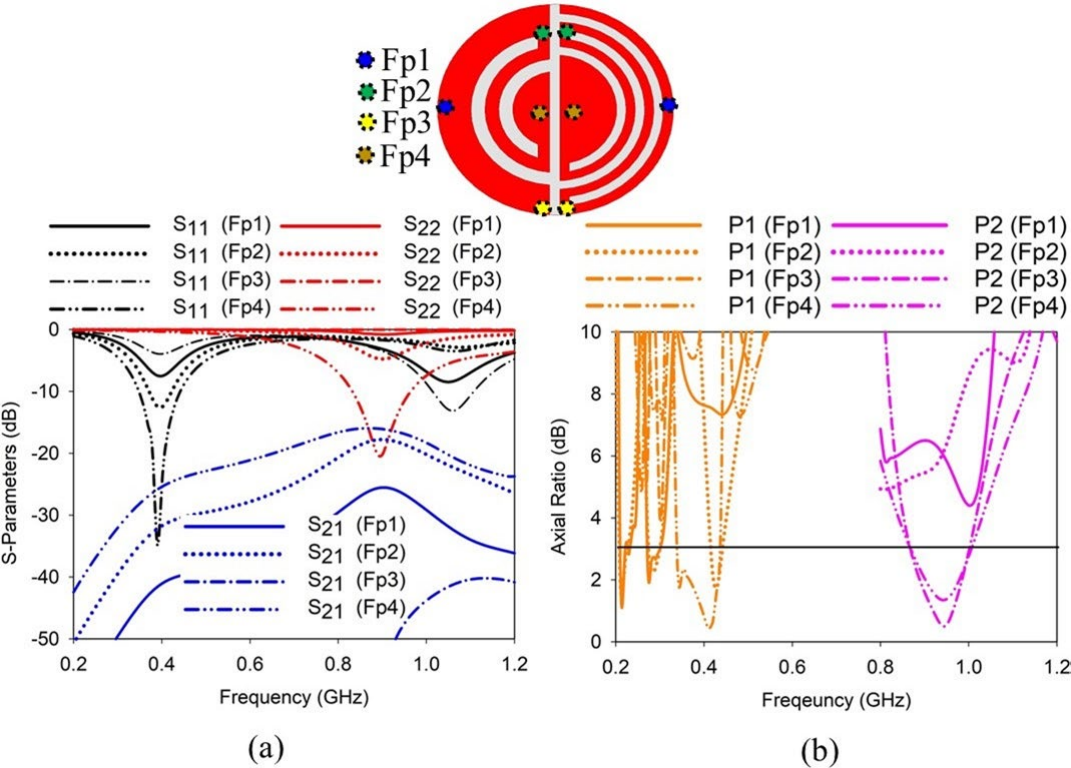
Effect of probe positions on (**a**) *S*-parameters, and (**b**) AR of the proposed SDCPI antenna.

The positions of the feeding probes play a critical role in determining the performance of the proposed SDCPI antenna. Figure 4 shows the effect of feeding probe positions on the *S*-parameters and AR of port 1 (P1) resonating at 402 MHz and port 2 (P2) resonating at 915 MHz. The matching of the lower resonance (402 MHz) improves as the probe moves from Fp1 to Fp4, giving the best results at FP4 as shown in Fig. 4a. The mutual The AR of P1 is also greatly affected by the probe position, giving AR < 3 dB in the band of interest for Fp4, as depicted in Fig. 4b. However, the upper resonance (915 MHz) shifts considerably to the lower frequency band along with improved matching as the probe changes location from Fp1 to Fp4. The optimum results are obtained at Fp4 as indicated in Fig. 4a. Similarly to P1, the AR of P2 is also influenced by the probe position, resulting in AR < 3 dB in the band of interest at Fp4, as shown in Fig. 4b. The mutual coupling between the ports also alters with the feeding probe position, and at Fp4, the mutual coupling between the ports is less than − 18 dB in the bands of interest, cf. Figure 4a.

#### Effect of shorting pin position

**Fig. 5:**
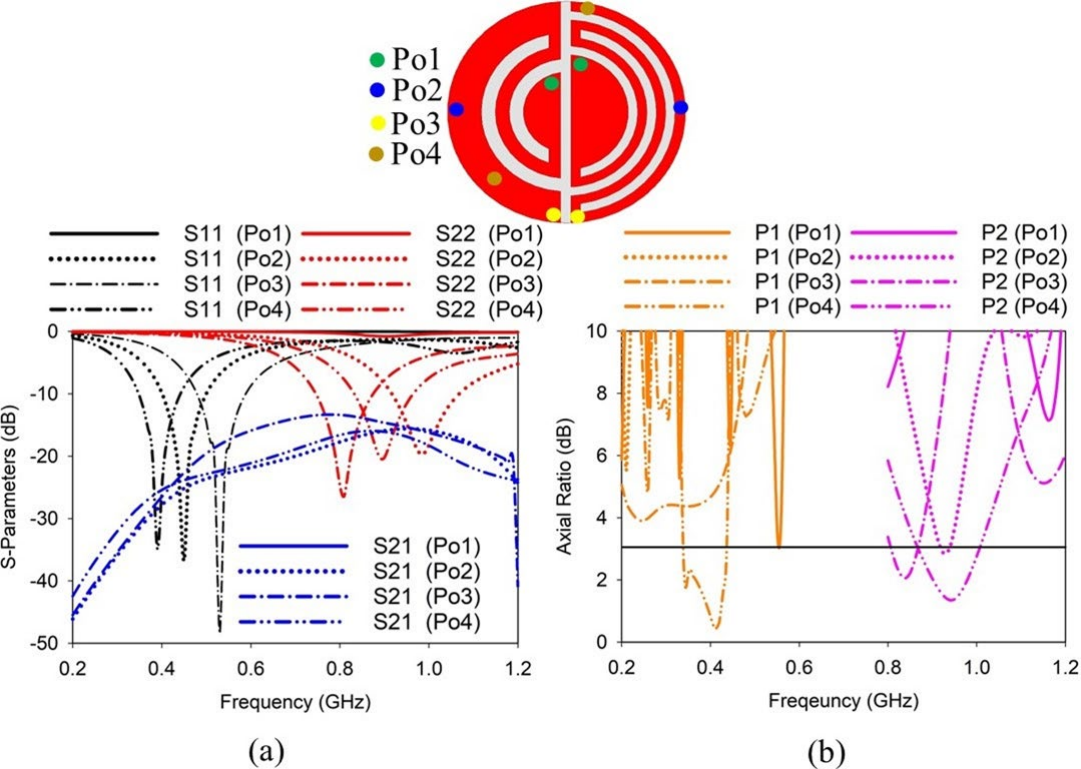
Effect of shorting pin position on (**a**) S-parameters, and (**b**) AR of the proposed SDCPI antenna.

The shorting pin positions are other important parameters deciding the performance of the presented SDCPI antenna. By varying the shorting pins from position Po1 to Po4, the performance at both ports (P1 and P2) in terms of *S*-parameters and AR varies significantly. As shown in Fig. 5a, when the shorting pins position changes from Po1 to Po4, the lower resonance (402 MHz) shifts considerably, with no resonance at Po1 and best matching at Po4. Likewise, the second resonance at 915 MHz also shifts considerably when shorting pins move from Po1 to Po4, with Po1 resulting in no resonance and Po4 resulting in excellent matching at around 915 MHz as depicted in Fig. 5a. Furthermore, the AR performance of both ports (P1 and P2) is highly sensitive to the variation in the shorting pin positions, as shown in Fig. 5b. When the shorting pins move from Po1 to Po4, the AR performance of both ports varies with Po4, resulting in AR < 3 dB for P1 and P2, as indicated in Fig. 5b. The mutual coupling between the ports varies with the shorting pin positions, with Po1 resulting in mutual coupling lower than − 70 dB and Po4 resulting in mutual coupling lower than − 18 dB in the bands of interest, as shown in Fig. 5a.

#### Effect of WCE components on the proposed SDCPI antenna

**Fig. 6:**
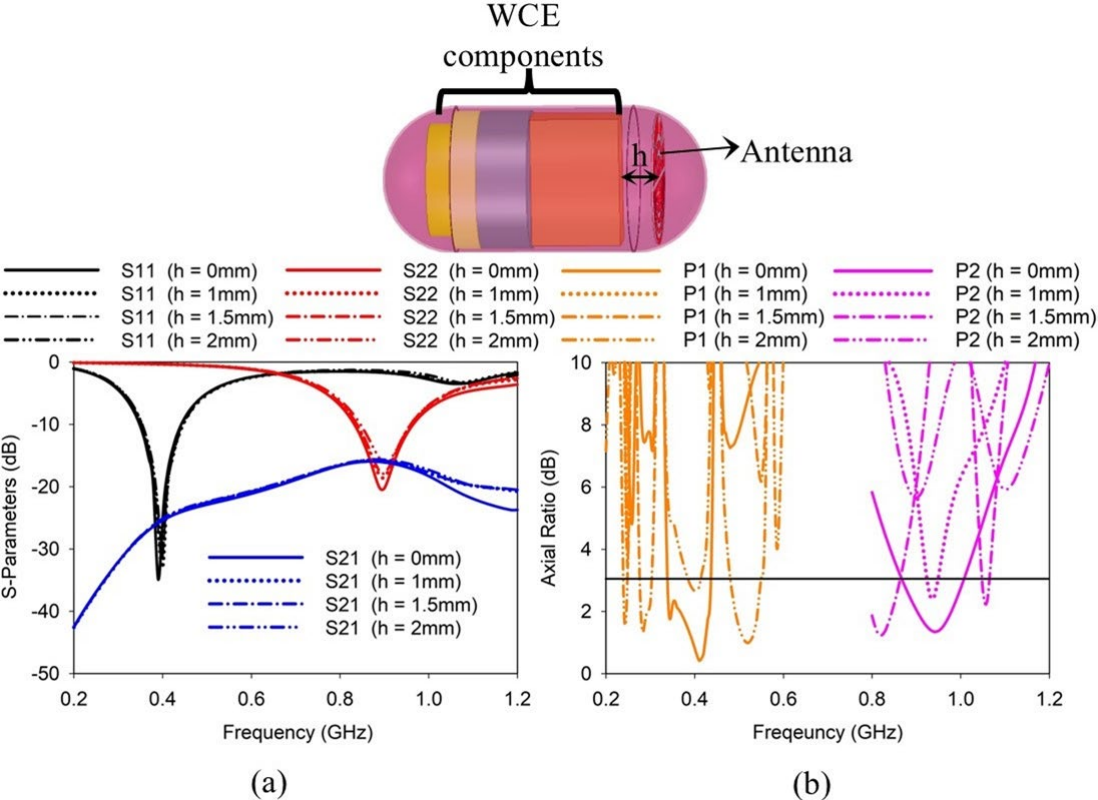
Effect of WCE components on (**a**) *S*-parameters, and (**b**) AR of the proposed SDCPI antenna.

A WCE device contains many components such as LEDs, PCB, camera, and batteries. These components have a significant impact on the performance of an implantable antenna. The proposed SDCPI antenna is embedded in a WCE device. Thus, its performance evaluation regarding its placement in the WCE is studied. Figure 6 shows the performance evaluation of the proposed antenna by varying the distance *d* between the antenna and the WCE components. As shown in Fig. 6a, the *S*-parameter performance of the presented SCPI antenna remains unaffected by varying *d*, however, AR at both bands strongly depends upon the placement of the antenna, as shown in Fig. 6b. According to Fig. 6b, the best position for placing the proposed antenna inside the WCE device is at *d* = 0 mm, resulting in AR < 3 dB in the desired bands.

## Results and discussion

This section includes a detailed analysis of the properties of the proposed SDCPI antenna. The results are discussed in terms of essential performance factors such as *S*-parameters, radiation pattern plots, SAR, and link budget analysis. To validate the simulation outcomes, physical measurements of the antenna prototype were conducted as detailed in Section-II(b).

**Fig. 7:**
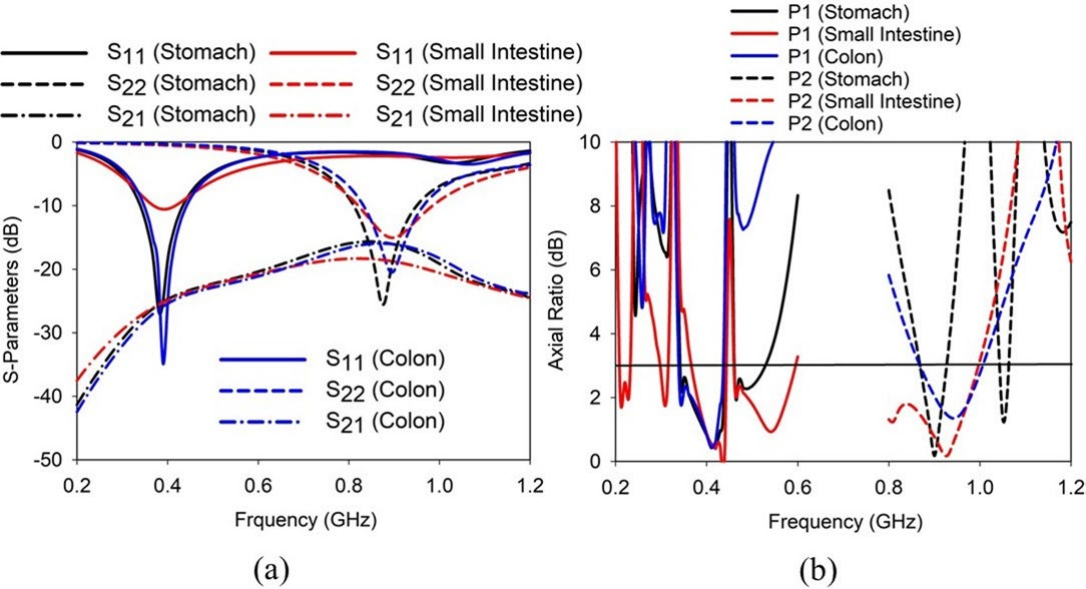
Simulated responses of the proposed SDCPI antenna in different GI tract tissues: (**a**) *S*-parameters, (**b**) AR.

The *S*-parameters (*S*_11_, *S*_22_, and *S*_21_) and AR plots of the proposed SDCPI antenna when placed in various GI tract tissues mimicking phantoms (stomach, small intestine, and colon) are presented in Fig. 7a, b. By inspecting Fig. 7a, it is evident that the reflection coefficients (*S*_11_ and *S*_22_) at port 1 (P1) and port 2 (P2) remain stable in the stomach and colon, providing a sufficient − 10 dB impedance bandwidth around 402 and 915 MHz, respectively. However, port 1 and port 2 reflection coefficients in the small intestine exhibit reduced impedance matching because of high conductivity of the small intestine, as presented in Fig. 7a. The mutual coupling between the ports remains almost constant in all the phantoms. The AR plot shown in Fig. 7b shows that the proposed SDCPI antenna provides AR < 3 dB in a wideband around 402 MHz (P1) and 915 MHz (P2), respectively, for all GI tract phantoms. Minor variations in the axial ratio between the stomach, small intestine, and colon phantoms are observed. These variations are primarily attributed to differences in dielectric constant and conductivity (Table 1), which influence the phase velocity and current distribution on the radiating patches. However, in all cases, the AR remains below 3 dB within the intended MICS, lower ISM, and upper ISM bands, confirming stable CP performance across the GI tract. These results confirm the robustness of the suggested SDCPI antenna for use in the whole GI tract.

**Fig. 8:**
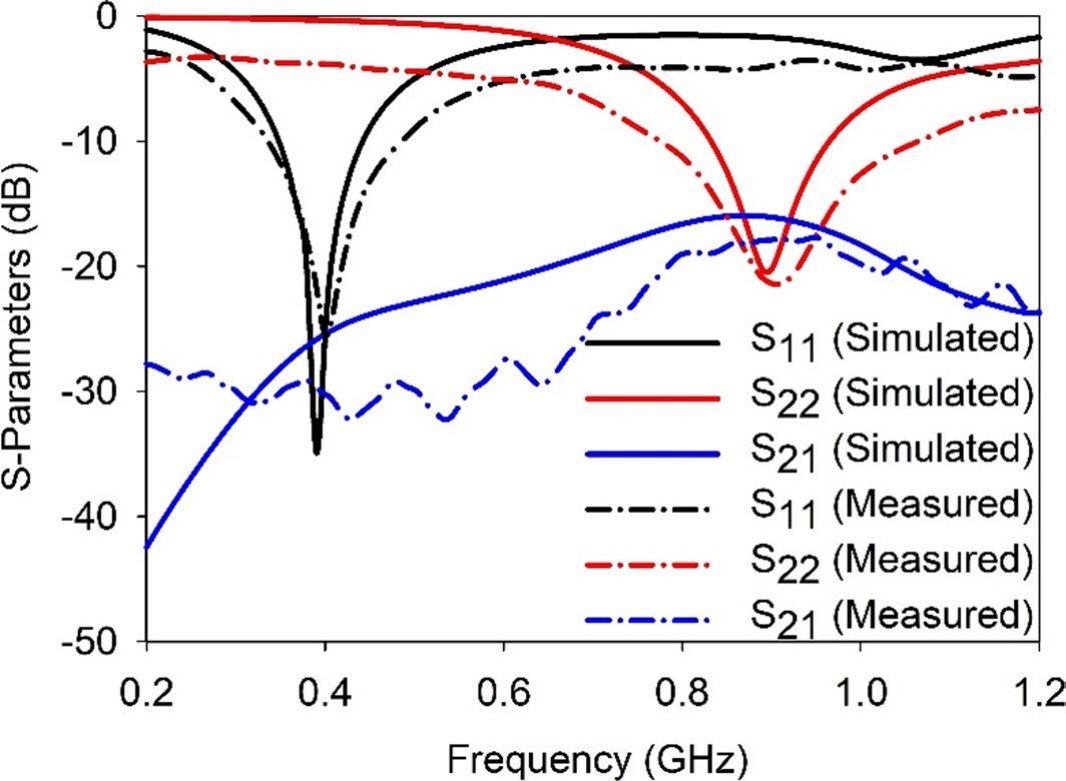
Simulated and measured S-parameters of the proposed SDCPI antenna.

The simulated (in colon) and measured *S*-parameters at ports P1 (*S*_11_) and P2 (*S*_22_) are shown in Fig. 8. In simulation, the proposed SDCPI antenna was resonating at 402 MHz (*S*_11_) and 915 MHz with − 10 dB impedance bandwidths of 92 MHz (348‒440 MHz) and 128 MHz (833‒961 MHz), respectively. The fractional bandwidths (FBW) offered are 23.3% and 14.2% at the respective bands. Furthermore, the measured resonances offered by the proposed SDCPI antenna were found to be 404 MHz and 920 MHz with − 10 dB bandwidths of 151 MHz (333‒484) and 273 MHz (782‒1055 MHz) and FBWs of 36.9% and 29.7% at the respective bands. The simulated (measured) isolation between the ports at 402 and 915 MHz bands is better than 23 dB (30 dB) and 18 dB (20 dB), respectively. Based on these results, it is confirmed that the proposed SDCPI antenna effectively covers the 402 MICS, 433 ISM, and 915 ISM bands for implantable applications.

**Fig. 9:**
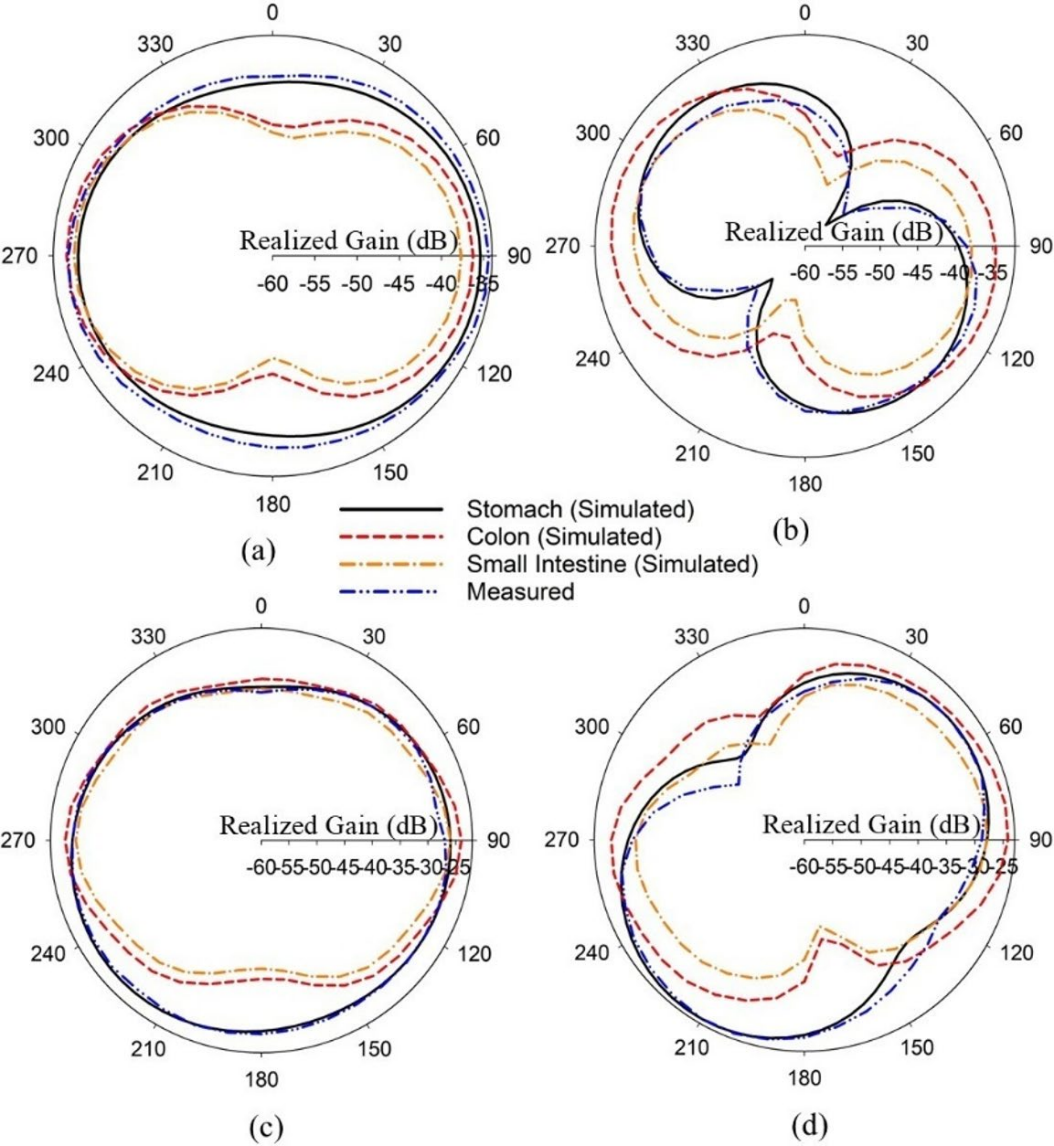
Simulated and measured radiation patterns in different phantoms: (**a**) E plane (402 MHz), (**b**) H plane (402 MHz), (**c**) E plane (915 MHz), (**d**) H plane (915 MHz).

The radiation patterns of the proposed SDCPI antenna were measured according to the arrangements discussed in Section II(b). The simulated and measured radiation patterns in the two principal planes (φ = 0° and φ = 90°) inside stomach, colon and small intestine are presented in Fig. 9a,b at 402 and Fig. 9c, d at 915 MHz. The measured radiation patterns are in close agreement with the simulated ones. In the E-planes at 402 and 915 MHz, the radiation patterns are nearly isotropic (Fig. 9a,c), while in the H-planes, the radiation patterns (Fig. 9b,d) are directional. Inside the stomach phantom, the peak simulated (in stomach) and measured gain at 402 and 915 MHz is − 38 dBi and − 36 dBi and − 26 dBi and − 25 dBi, respectively. Minor variations in beamwidth and sidelobe levels occur due to differences in tissue dielectric loading and attenuation, with the small intestine showing slightly narrower beams due to higher conductivity. As the SDCPI antenna is to be implanted inside the GI tract, these radiation characteristics allow complete coverage in one plane and the directed coverage in the other plane, resulting in the establishment of a reliable link between the proposed antenna and the outside station.

**Fig. 10:**
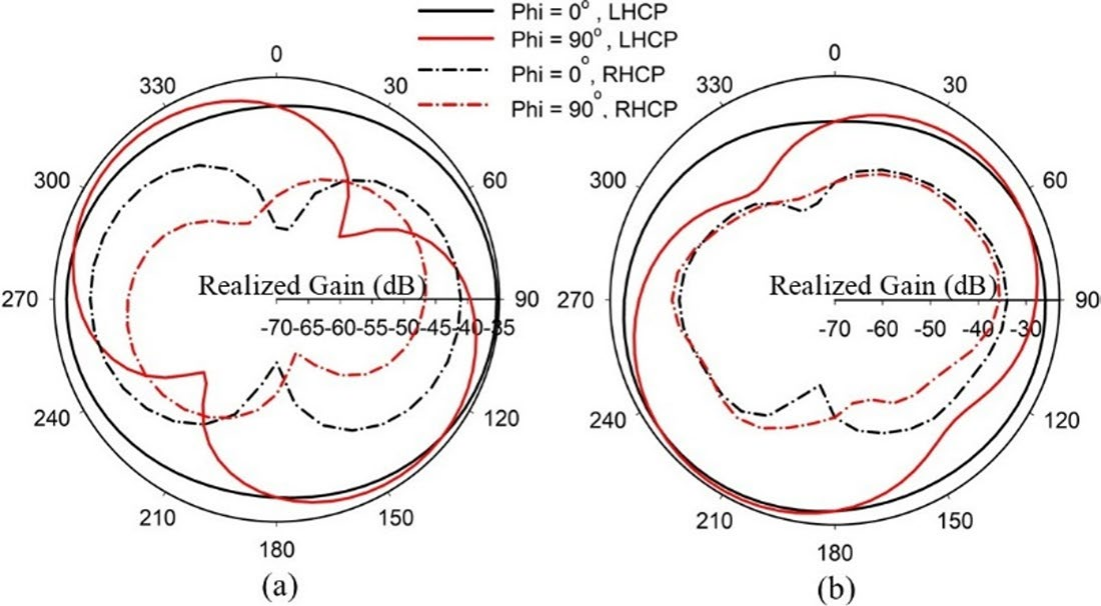
Simulated LHCP and RHCP radiation patterns: (**a**) 402 MHz and (**b**) 915 MHz.

The proposed SDCPI antenna maintains dual circular polarization: LHCP at 402 MHz (Port 1) and RHCP at 915 MHz (Port 2). To evaluate polarization purity, co- and cross-polarization radiation patterns were extracted as depicted in Fig. 10. The results show that the co-polarized component dominates the radiation, with cross-polarization at least 10–15 dB lower in the main beam direction, thereby validating the CP characteristics and purity of the antenna across the intended frequency bands.

**Fig. 11:**
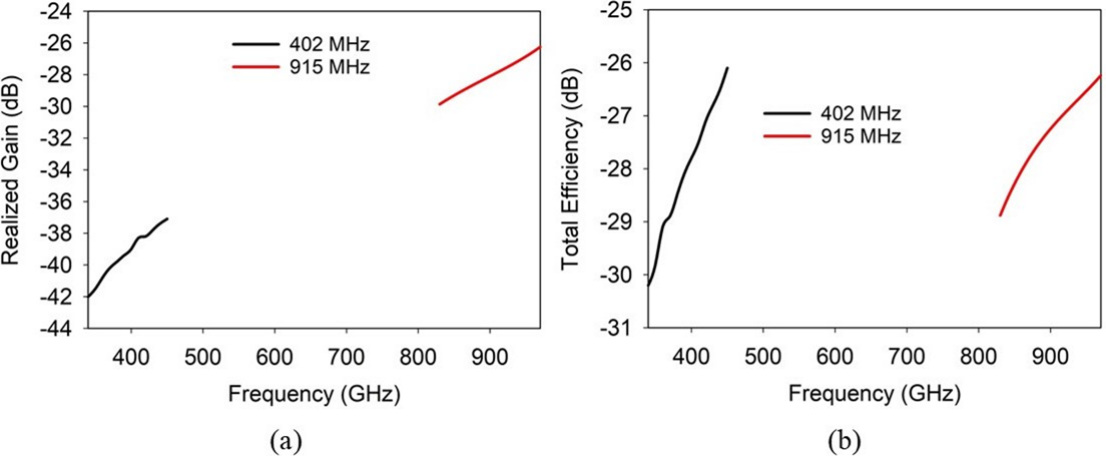
Proposed SDCPI antenna (**a**) realized gain and (**b**) total efficiency.

The simulated realized gain and total efficiency in the bands of interest of the suggested SDCPI antenna are presented in Fig. 11. The peak realized gains in the lower band (348 ‒ 440 MHz) is increasing gradually with frequency at peaks at 440 MHz to −36 dBi. Likewise, the peak realized gain in the upper band (833 ‒ 961 MHz) increases gradually with frequency with a maximum of −23 dBi at 961 MHz. These low gain values are primarily due to the strong dielectric loading and high conductivity of biological tissues, which cause significant attenuation for deeply implanted antennas. Furthermore, the total efficiency varies from −30.8 dB to −26.3 dB in lower band (348 ‒ 440 MHz) and from −29.7 dB to − 26.1 dB in the upper band (833‒961 MHz).

**Fig. 12:**
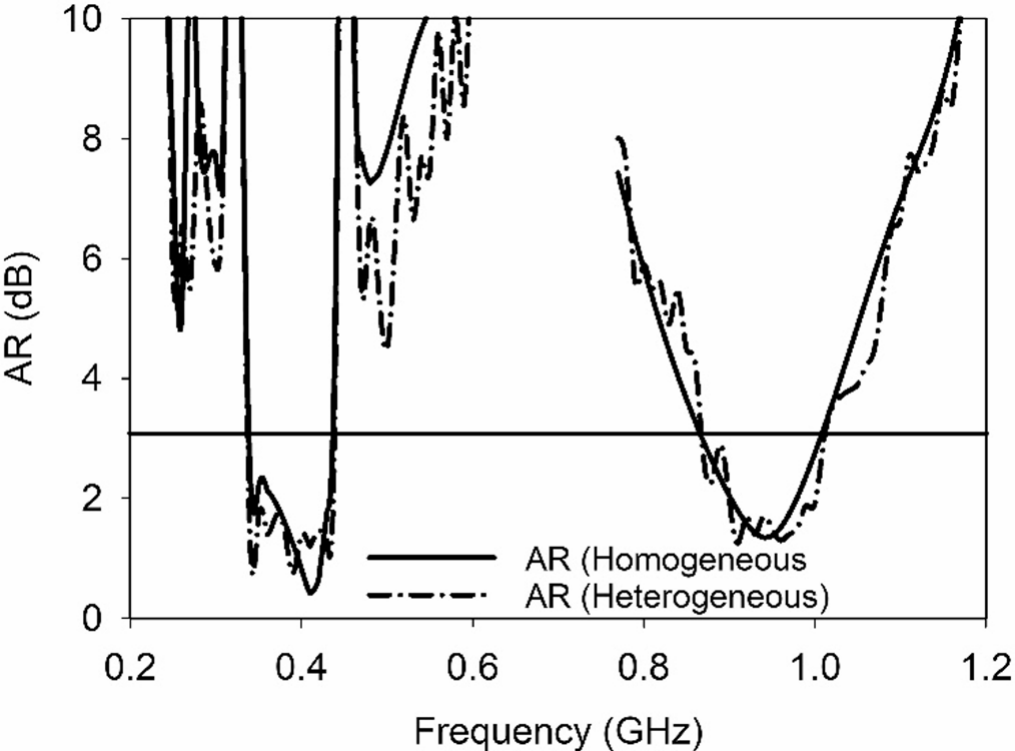
AR of the proposed SDCPI antenna in homogeneous and heterogeneous phantoms.

The simulated ARs in homogeneous and heterogeneous phantoms (stomach) of the proposed SDCPI antenna are presented in Fig. 12. It is evident that the ARs results consistently show AR < 3 dB across the target MICS and ISM bands, confirming stable circular polarization. The measured radiation patterns further support this simulated CP behavior.

**Fig. 13:**
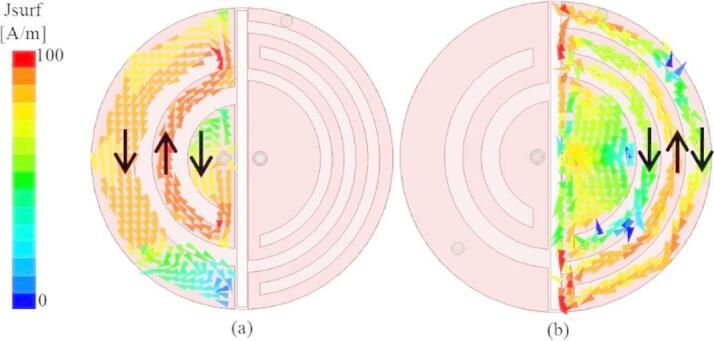
Surface current distribution (**a**) when port 1 (433 MHz) and (**b**) when port 2 (915 MHz) is active.

The proposed SDCPI antenna achieves inter-port isolation exceeding 20 dB without the use of conventional decoupling structures such as neutralization lines or EBGs. This high isolation is primarily attributed to (i) the orthogonal current distributions on the two semi-circular radiators, which minimize direct electromagnetic coupling as clear from the figure given below (Fig. 13). It is clear from Fig. 13a that when port 1 (433 MHz) is active the induced current on the inactive patch follows in opposite directions thereby canceling the effect of each other. Similarly, when port 2 (915 MHz) is active (Fig. 13), the induced current on the other patch also flows in orthogonal fashion resulting in field cancelations and achieving isolation better than 20 dB. (ii) the natural frequency separation between the ports (MICS/lower ISM for Port 1 and upper ISM for Port 2), which reduces mutual interaction.

**Fig. 14:**
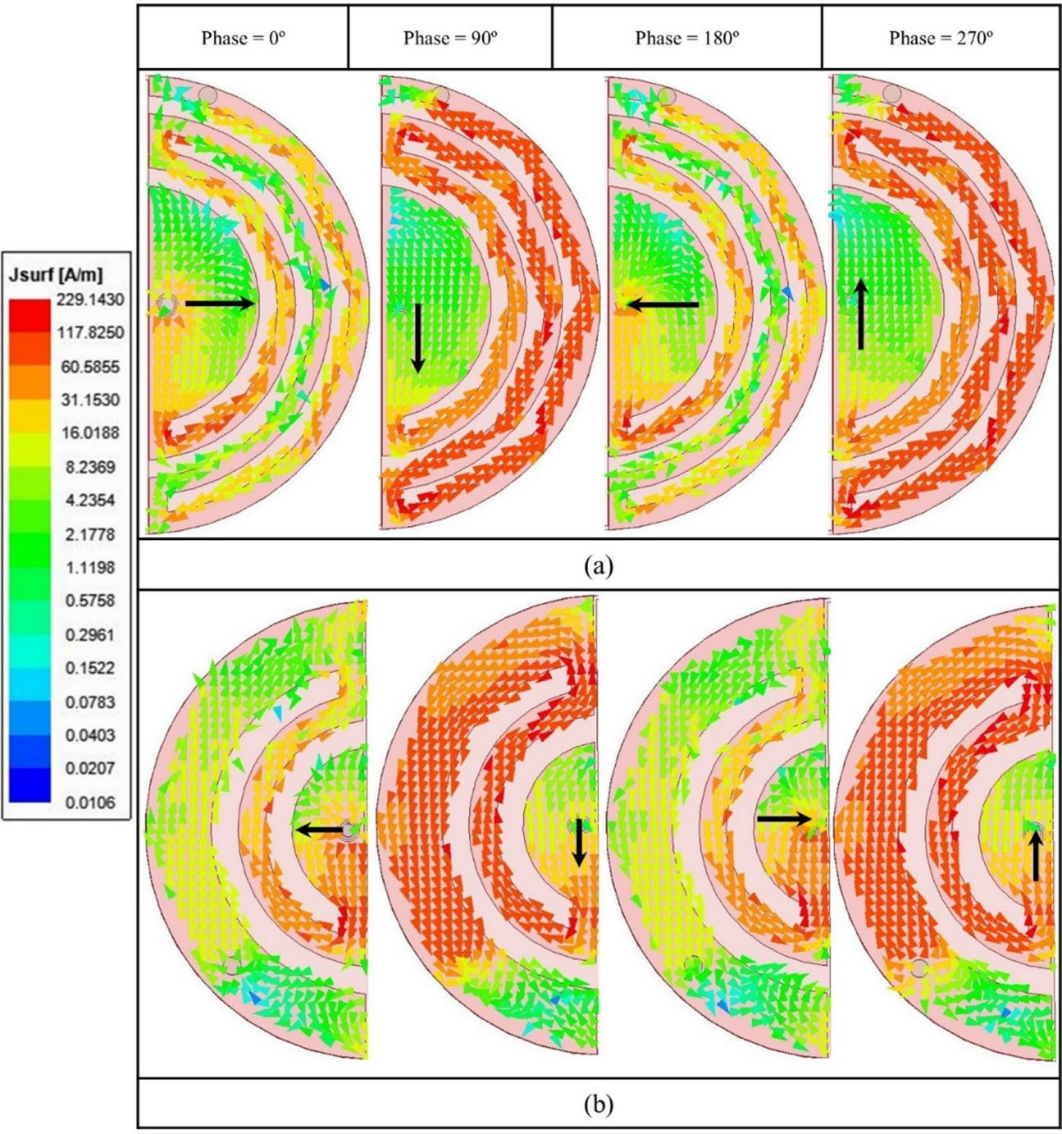
Current distribution on (**a**) patch 1(402 MHz), and (**b**) patch 2 (915 MHz).

The proposed capsule-integrated SDCPI antenna is composed of two semi-circular radiators with slots etched on both. These circular slots cause the E-fields to swirl around, resulting in CP behavior at 402 and 915 MHz. As can be observed in Fig. 7b, the SDCPI antenna exhibits CP performance when it is implanted inside various GI tract tissues (stomach, small intestine, and colon) at 402 and 915 MHz bands (with wide AR bandwidths at these frequencies). The surface current distribution (at different excitation phases) on both the radiating patches of the discussed SDCPI antenna is presented in Fig. 14a, b at 402 and 915 MHz, respectively. It is observed from Fig. 14a that at 402 MHz, the surface current rotates in the clockwise direction when the excitation phase changes from 0° to 270°, causing the patch to produce LHCP EM waves. When the excitation phases are 0° and 180°, most of the current concentrations are on the feeding point (flowing in opposite direction), while for excitation phases of 90° and 270°, the current concentrations are on outer slots with oppositely directed flow. Likewise, at 902 MHz (Fig. 14b), the current rotates in a counterclockwise direction when the excitation phase changes from 0° to 270°, resulting in RHCP EM waves generation. Furthermore, when excitation phase variations are 180° out of phase (0° and 180°), most of the current is concentrated on the feed with the oppositely directed flow. Similarly, for 90° and 270° excitation phases, the current is concentrated on the outer slots with opposite directions of flow. The combined influence of the current distributions across different phases resulted in the generation of a circularly polarized (CP) wave at 402 and 915 MHz.

**Fig. 15:**
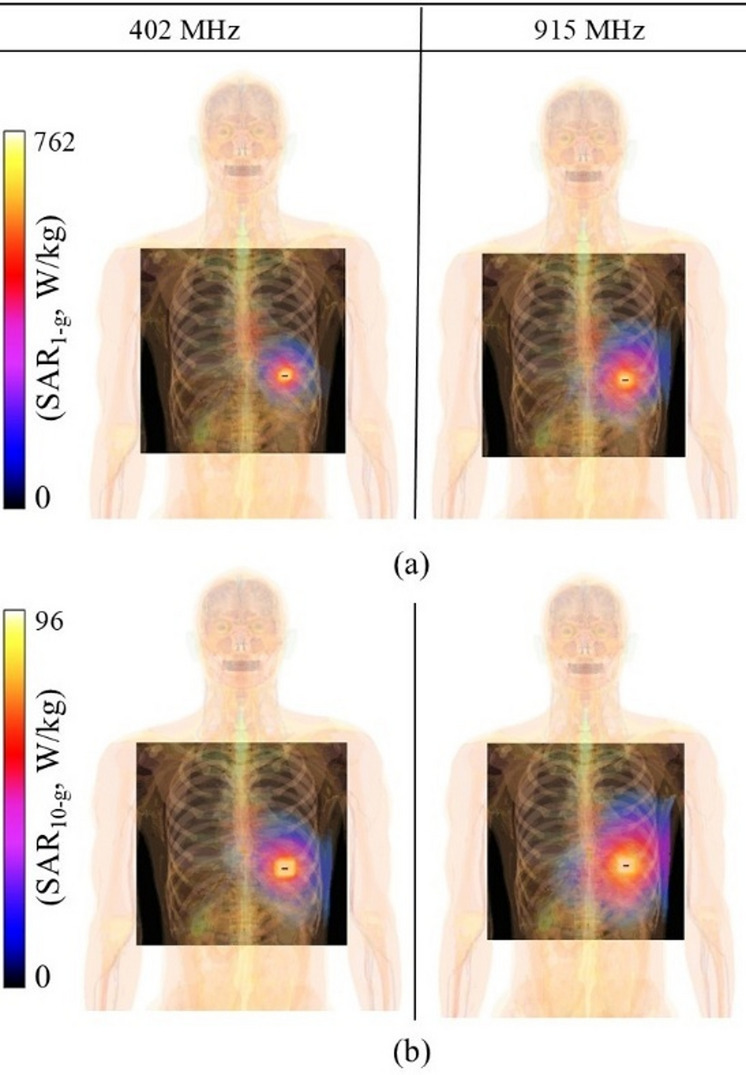
SAR distribution (**a**) 1-g and (**b**) 10-g at 402 and 915 MHz.

The presented capsule-integrated SDCPI antenna is suggested for WCE applications and is intended to communicate with an external receiver from within the GI tract. During this communication, EM waves will propagate through the GI tract and the human body, causing damage to the tissue if certain criteria are not strictly observed. Hence, it is crucial to study safety considerations of the proposed SDCPI antenna in terms of SAR and maximum allowable input power. The IEEE (IEEE C95.1-1999, IEEE C95.1-2005) standard suggests that the SAR value for 1 gram and 10 g of tissue should not exceed 1.6 W/kg and 2 W/kg^7^, respectively. The SAR of the proposed SDCPI antenna was evaluated using Sim4life tool in the stomach at 402 and 915 MHz for both ports with a 1 W input power exciting both ports, as presented in Fig. 15. To ensure high accuracy, a structured rectilinear mesh was generated with a maximum step size of 0.05 mm and a geometry resolution of 0.02 mm. These mesh settings are suitable for high-resolution voxel-based simulations and enable precise SAR calculation. The peak 1-g and 10-g SAR values in the stomach at 402 and 915 MHz are 643 and 762 W/kg and 91.7 and 96.4 W-kg, respectively. Based on 1-g SAR, the maximum allowable power in the stomach at 402 MHz (Port 1) is 2.4 mW and at 915 MHz (Port 2) is 2 mW. According to the ITU-R RS.1346 standards, the maximum allowable input power to implantable devices is restricted to 25 µW (–16 dBm) to avoid interference with other devices^1^. If the input power to the proposed SDCPI antenna is scaled down to − 16 dBm (currently 1 W), the evaluated SAR values would fall within the IEEE safety standards. These observations make the proposed SDCPI antenna safe to use in the GI tract as a part of the WCE system.

The range of effective and robust communication link established between the proposed SDCPI antenna and an external receiver is examined by exploring the link margin (LM) analysis of the presented design. Various important factors, such as cable losses, the effects caused by shadowing, losses due to impedance mismatch, free space losses, and losses due to polarization mismatch, are considered while evaluating the link margin^17^. During the evaluation of the LM, a CP antenna was used as a receiving antenna (Rx) (recall that the proposed antenna has CP characteristics), which was acting as a transmitting (Tx) antenna. This resulted in fixing the polarization mismatch loss to 0.6 dB. Additionally, as both Tx and Rx antennas are well matched, the impedance mismatch losses are set to 1 dB. The other parameters used in the evaluation of the LM are listed in Table 2. The LM is evaluated by keeping the input power at − 16 dBm, as fixed by IEEE to avoid interference with other communication systems. The *L*_*M*_ is evaluated as^1^:

3 $$\:{L}_{M}={P}_{rc}-{P}_{req}$$

where *P*_*rc*_ and *P*_*req*_ are the received power and required power, respectively. *P*_*req*_ is obtained as:

4 $$\:{P}_{req}=\frac{{E}_{b}}{{N}_{0}}+K{T}_{0}+{B}_{rate}$$

where *k* is the Boltzmann’s constant, *T*_*o*_ is the temperature, *E*_*b*_*/N*_0_ is the ideal phase shift keying, and *B*_*r*_ is the bit rate which is taken as 78 Mbps (mostly suggested for WCE applications).

Likewise, the receiving power *P*_*rc*_ is computed as:

5 $$\:{P}_{rc}={P}_{Tx}+{G}_{Tx}{+G}_{Rx}-{Pol}_{loss}-{L}_{path}$$

where *P*_*Tx*_, *G*_*Tx*_ and *G*_*Rx*_ are the gains of the transmitting and receiving antennas, *Pol*_*loss*_ is the polarization loss factor, and *L*_*path*_ is the path loss evaluated as:

6 $$\:{L}_{path}=20{\text{log}}_{10}\left(\frac{4\pi\:d}{\lambda\:}\right)+10\gamma\:{\text{log}}_{10}\left(\frac{d}{{d}_{0}}\right)+{S}_{d}$$

where *d* is the separation between the transmitting and receiving antenna, $\:\gamma\:$ is the path loss exponent and *S*_*d*_ is the shadowing effect.

**Fig. 16:**
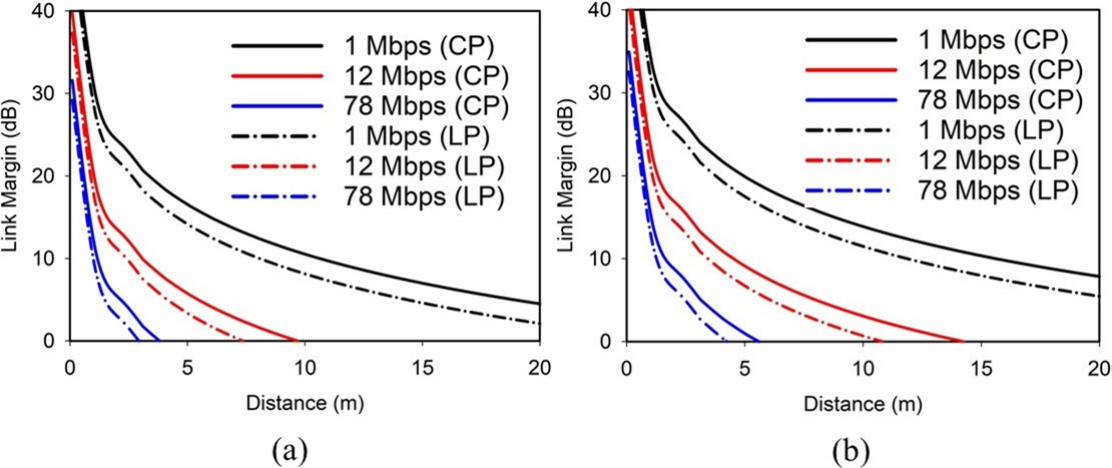
Link margin analysis of the proposed SDCPI antenna at 402 MHz and 915 MHz.

The LM of the suggested SDCPI antenna at 402 MHz (port 1) and 915 MHz (port 2) is evaluated based on (3) and presented in Fig. 16a, b, respectively. Two cases are considered; (a) when both the Tx and Rx antennas are CP and (b) when the Tx antenna is CP while the Rx antenna is linearly polarized (LP). The CP – CP link configuration between the implanted antenna and the external receiver results in a polarization mismatch loss of only up to 0.6 dB^26^. In contrast, an LP – LP configuration suffers from higher mismatch when there is misalignment between the implant and external antenna, and an LP – CP configuration typically suffers 3 dB loss. For both cases, the link margin at both resonances has been evaluated inside the small intestine phantom (worst case) by considering high data rates of 1, 12, and 78 Mbps, the last one being utilized by the next-generation BIDs. Figure 16a suggests that at 402 MHz for bit rates of 1, 12, and 78 Mbps the proposed SDCPI antenna offers reliable communication up to 25, 9.7, and 4.5 m (for case (a)), respectively while it maintains a coverage up to 20, 7.3, and 2.9 m for case (b). At 915 MHz, Fig. 16b shows that at the same bit rates, the proposed antenna offers coverage up to 28, 14.7, and 5.5 m (for case (a)) respectively while it maintains a coverage up to 24, 11, and 4.2 m for case (b). The increase in the coverage range at the upper frequency (915 MHz) is attributed to the increased gain of the proposed SDCPI antenna (-27.5 dB) as compared to the lower band (402 MHz), at which the gain is − 39.2 dB. These results suggest that the presented implantable antenna is highly effective in maintaining a reliable communication link with an external receiver when placed in the GI tract, making it a desired candidate for WCE applications.

**Table 2:**
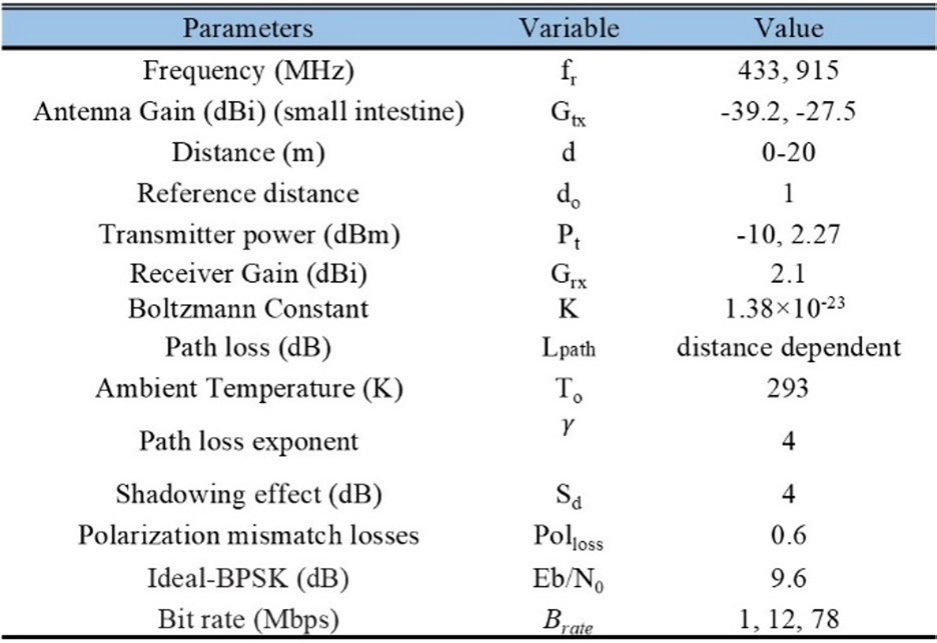
Link budget parameters.

**Table 3:**
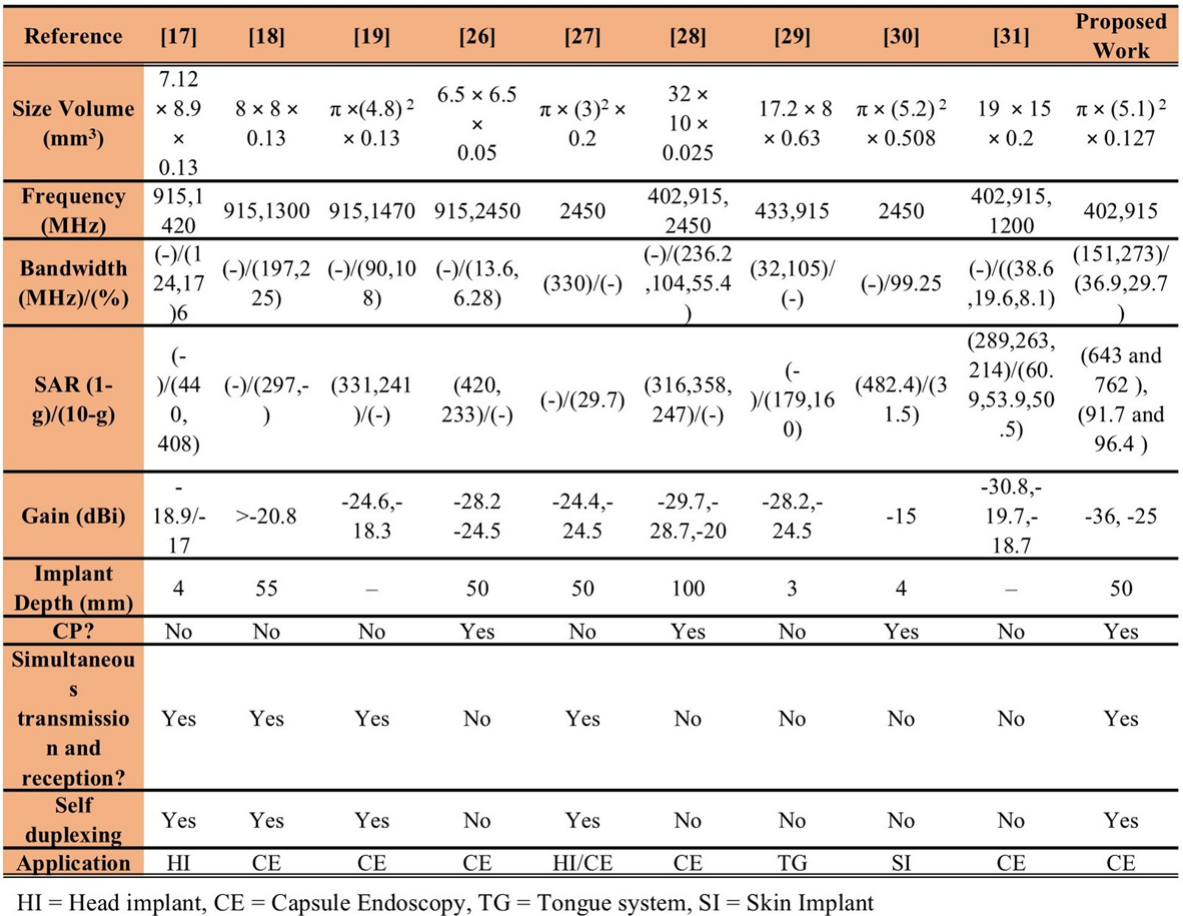
Comparison with other state-of-the-art implantable antennas.

A detailed comparison of the presented SDCPI antenna with the state-of-the-art implantable antennas is presented in Table 3. The proposed SDCPI antenna, when compared to similar self-duplexing implantable antennas^17,19,27^, offers operation in the MICS (402 MHz), lower ISM (433 MHz), and mid-ISM (915 MHz) bands, resulting in better tissue penetration and robust communication, which are highly desired for deep implantable applications. Additionally, all the self-duplexing implantable antennas^17,19,27^ are linearly polarized as opposed to the proposed SDCPI antenna, which is the only CP self-duplexing implantable antenna offering CP performance at both ports. The suggested SDCPI antenna is the most compact self-duplexing antenna in terms of electrical size as compared to the other implantable antennas presented in Table 3. Some of the conventional implantable antennas in Table 3^28–31^ offer CP performance with multiband operation in the MICS (402 MHz) and ISM (915, 2450 MHz) bands with a compact size, but would require an additional external multiplexer to separate the multiband for practical applications. This arrangement is associated with an additional space needed in the small-sized implantable device, resulting in serious issues and compromised performance. Based on the comparative analysis of the proposed SDCPI antenna with the state-of-the-art, it becomes evident that the presented solution is the first of its kind (to the best of our knowledge) proposed for WCE application, having MICS (402 MHz), lower ISM (433 MHz), and mid-ISM (915 MHz) bands of operation, has a very compact size, and offers CP operation at all the bands for both ports.

## Conclusion

In this study, a novel design of a dual-band, dual-port, self-duplexing circularly polarized implanted (SDCPI) antenna was presented. The proposed SDCPI antenna features a compact volume of π × (5.1)^2^ × 0.127 = 10.3 mm^3^ and offers dual-band CP performance at 402 (when port 1 is active) and 915 MHz (when port 2 is active) bands. Shorting pins and slotted radiation patches have been incorporated to achieve miniaturization and high port isolation of at least 10 dB at both operating bands. The proposed SDCPI antenna was integrated with a WCE capsule-type device, and rigorous simulations were carried out in the heterogeneous stomach, small intestine, the colon-type phantoms. The optimized antenna was fabricated, and measurements were taken in minced pork, showing good agreement with the simulated results. The SDCPI antenna offers peak realized gains of − 42 and − 34 dBi at 402 and 915 MHz, respectively. Additionally, SAR analysis was conducted by placing the capsule integrated into the SDCPI antenna inside realistic stomach, small intestine, and colon phantoms, showing excellent compliance with IEEE standards. The link budget analysis conducted confirms that the proposed antenna can effectively communicate with external base stations up to a minimum of 3 m (with a high data rate of 78 Mbps). These attributes make the proposed SDCPI antenna a suitable candidate for simultaneous multitasking applications in high data rate WCE applications.

## Data Availability

The datasets used and/or analysed during the current study are available from the corresponding author on reasonable request.
